# Report on the Changes in the Blood in Inflammation, and on the Nature of the Healing Process

**Published:** 1844-07

**Authors:** T. Wharton Jones

**Affiliations:** Lecturer on Anatomy, Physiology, and Pathology, at the Charing-Cross Hospital; Corresponding Member of the Imperial and Royal Society of Physicians and Surgeons of Vienna, &c.


### REPORT ON THE CHANGES IN THE BLOOD IN INFLAMMATION, AND ON THE NATURE OF THE HEALING PROCESS.

By T. Wharton Jones, F.R.S.
Lecturer on Anatomy, Physiology, and Pathology, at the Charing-Cross Hospital; Corresponding Member of the Imperial and Royal Society of Physicians and Surgeons of Vienna, &c.

### CHANGES IN THE BLOOD IN INFLAMMATION.

### I. CHANGE IN THE STAGNANT BLOOD.

§ 1. The red corpuscles stagnant within the vessels cease to be distinguishable individually, and appear as if fused together into a uniform red mass; interspersed throughout which (the frog being the subject of observation) the nuclei of the red corpuscles are still to be seen on careful examination.

§ 2. This change in the stagnant blood appears to have been first particularly referred to by Dr. Hastings. In the natural state of the blood, he observes, globules can be distinctly seen; but after inflammation has commenced the globular structure disappears, and the blood becomes redder.*

### II. EXPLANATION OF THE CHANGE IN THE STAGNANT BLOOD.

§ 3. The explanation of this change of the accumulated and stagnant blood-corpuscles which Emmert gives is this: The matter contained in the interior of the red corpuscles escapes with or without destruction of their walls, becomes mixed with the plasma still present in very small quantity, and thus forms a homogeneous looking mass, inclosing the nuclei which remain behind. That the substance contained within the blood-corpuscles escapes from them, is inferred partly from the disappearance of the blood-corpuscles, partly from the small quantity of plasma present, seeing that this although coloured would by no means be alone sufficient to form the homogeneous mass. Moreover that the colouring matter escapes from the red corpuscles is distinctly proved by the circumstance to be afterwards mentioned, that

* A Treatise on Inflammation of the Mucous Membrane of the Lungs: to which is prefixed an experimental inquiry respecting the contractile power of the blood-vessels, and the nature of inflammation. London, 1820, p. 95. Dr. Hastings's explanation of these alterations came as close to the truth as the state of knowledge of the blood and physiology in general at the time permitted. And it is to be remarked that Dr. Hastings points out a relation between these alterations in the blood and the debilitated action of the vessels, stating the occurrence of the change in the blood on its being received from healthy into debilitated vessels; and, *vice versâ*, its gradual reassumption of its natural appearance when, on the occurrence of resolution, the blood passed from these debilitated vessels into a large venous trunk, whose action was unimpaired. Though Dr. Hastings's work was published more than twenty years ago, it would still have done honour to the science of the present day, if it had now only appeared for the first time.

it exudes through the walls of the vessels, and tinges of a red colour the surrounding cellular tissue. Whether the wall of the blood-corpuscle remains during this process or is destroyed can scarcely be determined by direct observation. Still it does not appear that at the commencement of the change at least destruction takes place. It would rather seem that the disappearance of the previous form of the blood-corpuscles is owing to a transudation of their contents, and a coincident contraction of their walls. And this, because as will be mentioned when considering resolution of inflammation, it may happen that the blood mass which has but recently undergone the change is reconverted into blood-corpuscles, and passes again into circulation.*

§ 4. The reader who has already considered what the author of this Report has said in his 'Observations on the Blood,' and in his Report on the Nature of Inflammation, will readily detect the insufficiency of this explanation offered by Emmert, and will at once perceive that the apparent fusion of the red corpuscles is of the same nature as that which occurs in buffy blood, or in healthy blood, treated by the reagents already spoken of.

§ 5. The change in the appearance of the stagnant blood under consideration supervenes on stagnation more or less quickly. It may usually be observed to implicate the blood in the vessels first obstructed, and from thence to spread to the blood in the vessels more lately affected.

§ 6. This circumstance seems to show that the change is owing to some condition coming into operation subsequently to the stagnation. The apparent fusion of the red corpuscles being evidently of the nature above referred to, viz. owing to a change in their endosmotic state, to their having become somewhat distended, the condition on which it depends, it is to be inferred, is a change in the fluids by which the red corpuscles are surrounded.

§ 7. The change in the endosmotic state of the red corpuscles, and the change in the fluids by which the red corpuscles are surrounded, Henle endeavours to explain thus: "At a certain height of the inflammation absorption ceases, and the capillaries containing the stagnant blood-corpuscles are soon surrounded by a fluid which is thinner than blood plasma, and cannot but change the blood-corpuscles in the manner above mentioned. It is as if the blood-corpuscles having been separated from the plasma by filtration were treated with diluted serum." (Bericht, p. 129.)

§ 8. Undiluted serum has not, as is well known, the effect of causing much distension of the red corpuscles by endosmose, and when sufficiently diluted to cause much distension, it at the same time prevents the aggregation of the red corpuscles or disperses it if it has already taken place. (§ 107.)

§ 9. The distension and apparent fusion of the red corpuscles stagnant within the vessels being like what are observed in buffy blood out of the body, and the same as may be artificially produced by an increase of viscid matter and diminution of salts in the plasma, the author of this Report believes that the change is owing to the action of a plasma inspissated as regards its protein constituents, but deprived of a portion of its salts by exudation of serum. In fact, he believes the apparent fusion of the red corpuscles which supervenes on their stagnation within the vessels to be owing to the action of the plasma in that very condition to which, it was shown in the former Report, (§ 40,) Henle attributed the first stagnation of the red corpuscles, but, as the author endeavoured to show, erroneously. Instead of the first stagnation, Henle's arguments find their application, in explanation of the changes in the blood under consideration and that with full force.

§ 10. In the course of the endosmotic changes just described, while the red corpuscles receive into their interior a portion of the surrounding and altered plasma, they give out a part of their contents, especially the colouring matter. This passing out from the vessels with the exuded fluid, tinges the adjacent parenchyma.

§ 11. To this transudation of colouring matter from the blood in the vessels to the adjacent parenchyma, as also to the endosmotic changes in the red corpuscles, the variations in the tint of the redness of an inflamed part appear to be owing.

* Beiträge zur Pathologie und Therapie, Erstes Heft, p. 84. Bern, 1842.

§ 12. When stagnation has not continued long before resolution takes place, the red corpuscles though distended and apparently fused together appear to remain entire, and on being restored to the circulation, resume their former collapsed and isolated state. But if stagnation has already existed for any length of time before resolution occurs, and especially if some other event than resolution supervenes, it is very probable that some of the red corpuscles burst and dissolve. See § 89.

III. CHANGES IN THE GENERAL MASS OF BLOOD.

§ 13. Inflammation may arise without any previous change in the general mass of blood, and if limited in extent, may run its course without the supervention of any such change. As, however, in inflammation of any severity and extent, changes in the general mass of blood early show themselves, and as these changes when they occur, exert an important influence by reaction on the inflammation and its events, they here require some consideration.

§ 14. The most characteristic and important change which the general mass of blood in inflammation presents, is an increase in the quantity of fibrin, and a decrease in that of the red corpuscles. And in connexion with this change in respect of composition, there is the remarkable increase in the tendency of the red corpuscles to aggregate into rolls, when the blood is drawn from the body.

§ 15. In consequence of the decrease in the quantity of red corpuscles, blood drawn in a well-marked case of inflammation appears paler, thinner, and more fluid than natural. So much more fluid is it, that if a prick be made in the finger of the patient, the blood, instead of oozing slowly out in the form of a drop, flows out quickly, and spreads on the surface of the skin.

§ 16. In consequence of the increase in quantity of the fibrin and of the increased tendency of the red corpuscles, diminished in quantity, to aggregate together, blood drawn in inflammation presents the buffy coat, and that by the following process as formerly explained in this Review: "The minute process leading to the separation of the liquor sanguinis from the red corpuscles, the visible condition for the formation of the buffy coat, consists in an exaltation both of the rapidity and closeness with which the red corpuscles naturally aggregate into rolls, and these again into a sponge-work, thus squeezing out the liquor sanguinis from among the corpuscles, and allowing the *greater specific gravity of the latter to come more fully into play*, whereby the liquor sanguinis, which in such cases is in relatively greater quantity, collects at the top, and coagulating, gives rise to the buffy coat."*

§ 17. As excess of fibrin constitutes so striking a character of the condition of the blood under consideration, the late Dr. Simon of Berlin, distinguished it by *the name of hyperinosis*, (from ὑπερ and ἴς, ἰνος, flesh.)†

* For details on this head the reader is referred to the author's 'Observations,' in this Review, for Oct. 1842, and to a short paper in the Edin. Med. and Surg. Journal, for Oct. 1843. Before leaving the subject, however, the author of this Report begs to say that Dr. Williams, (Principles of Medicine, § 205, p. 96,) in representing and objecting to his views, as laid down in the 'Observations' just referred to, has altogether fallen into error. To guard against the erroneous supposition to which the observation of a thin stratum of blood under the microscope is apt to lead, viz. that the red corpuscles run less closely together into a mass in buffy than in healthy blood, the author of this Report thought it necessary, in his 'Observations,' to devote a long paragraph, p. 594. This Dr. Williams has entirely overlooked, and adduces the erroneous supposition in question as an objection to the author's comparison to a contracted sponge. Dr. W. moreover speaks as if the author had not taken into consideration the specific gravity of the red corpuscles, whereas it is distinctly referred to, not only in the extract above given but in two or three other places. It is curious to find how differently from the English author a foreigner represents the author's statements on this subject: "Wharton Jones," says Henle, (Bericht, &c. p. 120,) "legt besondern Werth auf das vermehrte specifische Gewicht des Blutkuchens. Indem nämlich die Säulen der Blutkörperchen zu einem viel dichteren Netzwerk sich zusammenfügen als im normalen Blut, sind die von Plasma, erfüllten Räume relativ kleiner und die ganze Masse wird schwerer."

† Besides inflammations, hyperinosis occurs in some other diseases, e. g. phthisis pulmonalis, and also physiologically in pregnant women.

An opposite condition of the blood, viz. diminution in the quantity of fibrin, the quantity of red corpuscles remaining the same, or the quantity of fibrin remaining the same, an increase in the quantity of red corpuscles, occurs in fevers, hemorrhagic diseases, plethora. To this Simon has given the name of *hypinosis*, from ὑπο and ἴς, ἰνος.

§ 18. The natural quantity of fibrin and red corpuscles in the 1000 parts of blood is :—

| *According to Simon.* | | *According to Lecanu, and adopted by Andral and Gavarret.* | |
|---|---|---|---|
| Fibrin . . . | 2·1 | Fibrin . . | 3·000 |
| Red corpuscles . . | 110·000 | Red corpuscles . . | 127·000 |

§ 19. The fibrin may in hyperinosis of the blood increase, according to Simon, to four times, according to Andral and Gavarret, to three and a half times the above quantities respectively admitted by them as natural. The red corpuscles, on the other hand, may diminish, according to Simon to one third, according to Andral and Gavarret, to one sixth of their natural quantity.

§ 20. The remarkable inverse proportion in the quantity of fibrin and red corpuscles in hyperinosis of the blood, is well illustrated by the two following tables, drawn out by Simon, the first from his own analyses, the second from those of Andral and Gavarret.*

Taking the quantity of the solid constituents of the blood at 100, the relation in respect of quantity between the fibrin and red corpuscles stands thus:

| | *Fibrin.* | *Red corpuscles.* |
|---|---|---|
| Healthy blood | 1 | 53 |
| Morbid blood | 1·4 | 43 |
| ” | 1·6 | 40 |
| ” | 1·7 | 40 |
| ” | 2·0 | 42 |
| ” | 2·0 | 39 |
| ” | 2·1 | 36 |
| ” | 3·0 | 28 |
| ” | 6·0 | 22 |

| | *Fibrin.* | *Red corpuscles.* |
|---|---|---|
| Healthy blood | 1·5 | 61 |
| Morbid blood | 2·5 | 60 |
| ” | 3·2 | 57 |
| ” | 4·1 | 57 |
| ” | 4·2 | 54 |
| ” | 4·8 | 52 |
| ” | 5·0 | 50 |

§ 21. In the ‘Observations’ on the Blood in this Review, for Oct., 1842, it was remarked that though blood on which a buffy coat afterwards forms, appears thinner than natural, this is not owing to any increased tenuity of the coagulable lymph, or

* The opposite inverse proportion in the quantity of fibrin and red corpuscles in *hypinosis* of the blood is illustrated by the following table reduced by Simon from the analyses of Andral and Gavarret. In the first series the proportion of fibrin and blood-corpuscles is that in the 100 parts of solid constituents of the blood. The second series is the same, only the quantity of fibrin is constantly given at 1·5, and the proportion of blood-corpuscles reckoned accordingly. By this means the increase of the latter is rendered more striking.

| | *First Series.* | | | *Second Series.* | |
|---|---|---|---|---|---|
| | Fibrin. | Red corps. | | Fibrin. | Red corps. |
| Healthy blood | 1·5 | 61 | Healthy blood | 1·5 | 61 |
| Morbid blood | 1·5 | 64 | Morbid blood | 1·5 | 64 |
| ” | 1·5 | 65 | ” | 1·5 | 65 |
| ” | 1·3 | 60 | ” | 1·5 | 69 |
| ” | 1·1 | 53 | ” | 1·5 | 72 |
| ” | 1·2 | 59 | ” | 1·5 | 74 |
| ” | 1·1 | 60 | ” | 1·5 | 81 |
| ” | 1·0 | 60 | ” | 1·5 | 90 |
| ” | 0·9 | 60 | ” | 1·5 | 90 |
| ” | 1·0 | 61 | ” | 1·5 | 91 |
| ” | 1·0 | 64 | ” | 1·5 | 96 |
| ” | 0·9 | 63 | ” | 1·5 | 105 |
| ” | 0·5 | 60 | ” | 1·5 | 180 |

more properly speaking, liquor sanguinis, but to a diminution in the number of red corpuscles. This circumstance of the liquor sanguinis not being attenuated in blood in the state of hyperinosis, though, in consequence of the diminution in the quantity of the red corpuscles, the quantity of solid constituents in the aggregate of the blood is diminished, and the blood as a whole therefore more watery, Henle has illustrated in a striking manner from the data furnished by the analyses of Andral and Gavarret, and of Simon.

22. Excluding from consideration the red corpuscles, and comparing the plasma of the blood in inflammation with that of the blood in health, the quantity of water is in the former case less than in the latter. Besides this diminution in the quantity of water in the plasma of the blood in inflammation, it appears that there is frequently a diminution in the quantity of salts, whilst the quantity of fat, albumen, and fibrin, is increased; the quantity of the latter in general to a greater amount than that of the former.

§ 23. This actual diminution in the quantity of water in the blood in inflammation, Henle thinks is produced by the exudation of serum, and, as was shown in the last Report, it is the inspissation of the plasma resulting therefrom, which he thinks is the condition which immediately determines the agglomeration of the slowly flowing corpuscles, and their subsequent stagnation.

§ 24. The objection, however, was raised to this view, that stagnation precedes the exudation of serum. And in the present Report, it has been shown, (§ 9,) to be probable that it is the changes which take place in the blood subsequently to its first stagnation, which are produced by the plasma inspissated, as regards its protein constituents, but deprived of a portion of its salts.

§ 25. Hitherto the protein compound coagulable spontaneously, found in buffy blood, has been called fibrin, but according to Mulder's recent researches,* it would appear that the buffy coat does not consist of true fibrin, but is composed of a *binoxide* of protein, which is insoluble in boiling water, and a *tritoxide* which is soluble. These oxides Mulder comprehends under the name of *oxyprotein.*

§ 26. Is the quantity of colourless corpuscles of the blood changed in inflammation in the stage under consideration? That the quantity is increased is very generally maintained principally on the following grounds:

1st. The accumulation of colourless corpuscles within the vessels of an inflamed part.

2d. The large quantity of colourless corpuscles contained in the liquor sanguinis, which in blood drawn in inflammation, rises to the top to form the buffy coat.

3d. Mr. Gulliver's observations in the 'Philosophical Magazine,' for September, 1838, have been especially adduced by Dr. Carpenter, as being in his opinion at least, satisfactory proofs that "the usual amount of colourless corpuscles in the blood is very much increased at the time when an increase of fibrin is taking place, that is in the inflammatory condition."†

§ 27. In regard to the accumulation of colourless corpuscles within the vessels of an inflamed part, it was shown in the Report on Inflammation, § 35, in the last Number of this Review, not to be any indication of an increase in the quantity of colourless corpuscles in the general mass of blood. It is in fact no more an indication of this than the occurrence of a crowd in the street before one's window, can be reckoned an indication that the number of inhabitants of London is amazingly increased.

* Ueber die Oxydationsproducte des Proteins im thierischen Organismus; in Wöhler and Liebig's Annalen der Chemie und Pharmacie, Band xlvii. Heidelberg, 1843.

† Report on 'Cells,' in this Review, for Jan. 1843. M. Gendrin has been referred to also as an authority that the colourless corpuscles are much increased in number in inflammation, but no reference is made to the work where M. Gendrin has made a statement to such an effect. The author of this Report has not been able to find it in the 'Histoire des Inflammations.' Indeed when M. G. wrote this work (1826) he did not know of the existence of colourless corpuscles farther probably than this, viz. that the corpuscles which, in the frog, he took for pus-corpuscles, into which he supposed the red corpuscles had become converted, were most probably colourless corpuscles.

§ 28. On the large quantity of colourless corpuscles in the buffy coat,* considered as an indication of an increase in their quantity in the general mass of blood, it was remarked in the observations on the Blood, in this Review for October, 1842, that without denying that the colourless corpuscles may be more numerous in buffy than in healthy blood, it is probable for the reasons given that they are actually not so very much so as might at first have been supposed. And this especially as in microscopical examinations of newly-drawn blood, which afterwards became buffed, any very marked increase over what occurs in health, in the number of colourless corpuscles, was not observed.

§ 29. As to the proofs derived from Mr. Gulliver's observations, it is to be remarked: Mr. Gulliver's object in his paper appears to have been simply to establish the fact of the existence in the blood he examined of corpuscles, which he considered pus-globules; not at the time being aware of the existence naturally of the colourless corpuscles of the blood. His expressions do not point to any great numbers. Had healthy blood been treated in the same way, colourless corpuscles would also have been "detected," "numerous colourless corpuscles would have been observed," "many would have been seen," "several would have been found," &c., and certainly healthy blood examined without any preparation will not "rarely' but *always* present colourless corpuscles.

§ 30. But supposing that the corpuscles referred to by Mr. Gulliver, had been *all* colourless corpuscles, (which, however, Mr. Gulliver does not seem to admit,) and that had their quantity, instead of being vaguely indicated, been accurately determined, and had they been found by comparison with the quantity of colourless corpuscles in healthy blood actually increased, as Mr. Gulliver's subsequently published statements warrant the supposition they would have been found to be,† do Mr. Gulliver's cases of 1838, and his subsequently published ones, come under the head of inflammation proper, at present under consideration, the stage of inflammation at which an increase of fibrin, hyperinosis, is taking place? No: they were cases for the most part of suppurative inflammation, under which the subjects of them, with two or three exceptions, sank. It is also to be remarked that, with these exceptions, it was only after death that the blood was examined.‡

§ 31. The inference from what has been now said is this: In cases of recent inflammation, i. e. when hyperinosis is taking place, it has not yet been shown that there is any very great change in the quantity of colourless corpuscles, but when the inflammation has already existed some time, and as Mr. Gulliver says, especially when attended by suppuration, there may be a marked increase in the quantity of colourless corpuscles.

### IV. EXPLANATION OF THE CHANGES IN THE GENERAL MASS OF BLOOD.

§ 32. *Source of the fibrin in hyperinosis of the blood.* Considering that it is in the blood that fibrin first appears in any considerable quantity, and endowed with the strong and peculiar tendency to become organized, and considering that the red corpuscles are principally if not wholly composed of protein matter, the author of this Report suggested in his 'Observations on the Blood,' already often referred to, that the more peculiar object of the elaboration supposed to be performed by the red corpuscles in their capacity of glandular cells might be the conversion of one

* The buffy coat in anæmia is not here referred to; though this may contain a very large quantity of colourless corpuscles, it contains very little fibrin.

† In his Appendix to Gerber's General Anatomy (1840), Mr. Gulliver speaks of the pus-globules found in the above experiments and cases thus: In the inflammatory diseases, especially when attended by suppuration, whitish globules, which I have elsewhere described as those of pus, may be found in unusual numbers in the blood, (p. 20.) And again, in the Philosophical Magazine for Sept. 1842, in a paper on the pus-like globules of the blood, Mr. Gulliver says: "In some of my earlier observations these two varieties of globules (colourless corpuscles of the blood and pus-globules) were doubtless confounded; and their similarity is often so close that it may well be questioned whether there is any essential difference between them in many cases, although it is difficult to avoid attributing to the effects of disease the unusual abundance of pus-like globules in the blood of patients labouring under numerous inflammatory distempers."

‡ Andral (Essai d'Hématologie Pathologique, p. 113 et seq.) gives three similar cases.

protein compound into another—albumen into fibrin—a less into a more highly organizable proximate principle.

§ 33. He was further corroborated in this view by reflecting on the remarkable inverse relation, as regards quantity, between the fibrin and red corpuscles of the blood in disease; for, according to the theory of secretion, on the principle of which the view depends for its foundation, the secreting cells are, with their contents, resolved into the secreted fluid; and therefore, as the secreted fluid increases, the secreting cells must decrease in quantity, to be replaced by newly-formed ones.

§ 34. The inference in regard to the source of the fibrin in hyperinosis of the blood from all this was obvious, viz. that the augmentation of fibrin is at the expense of the red corpuscles which have disappeared, these in consequence of increased action, having been more quickly and in greater quantity resolved into fibrin than in health, and that in a ratio disproportionate to the production of new red corpuscles.

§ 35. This view of the production of fibrin the author of this Report enunciated as his own, in his Observations on the Blood, in this Review, for October, 1842, and in his Report on Inflammation, in the last Number, he claimed it, protesting against the award of it which had been made to Professors Wagner and Henle, by Drs. Carpenter and Williams. Since then the late Dr. Simon's 'Physiologische und pathologische Anthropochemie' having come into his hands, he finds that Dr. Simon had already enunciated a similar view.*

§ 36. After adducing various arguments in support of the opinion, that fibrin is formed from the red corpuscles, Dr. Simon (p. 68) goes on to say: The formation of fibrin by metamorphosis of the red corpuscles is, however, shown much more clearly—I might say convincingly—from my researches on the blood. These show that the quantity of fibrin is, with rare exceptions, always in a pretty definite inverse proportion to the quantity of red corpuscles—the more red corpuscles blood contains, the less fibrin—or, the more fibrin, the fewer red corpuscles. Admitting that the fibrin is formed from the red corpuscles, this phenomenon is simply explained; as when there is a greater consumption of red corpuscles, the quantity of fibrin in the plasma must increase.

§ 37. Which of the component parts of the red corpuscles, he then goes on to ask, is that which is most likely expended in the production of fibrin—that most important constituent of the plasma? It can scarcely be *globulin*, he answers, as it amounts to from four to ten per cent. of the blood; and, being a protein compound, is chemically so closely allied to fibrin, that, admitting a conversion of globulin into fibrin, we should expect to have a much greater quantity of fibrin in the blood than is found in it. It is, he continues, still less likely to be the *hematin* of the red corpuscles which is expended in the production of fibrin. The object of the hematin rather appears to be, to promote and maintain the independent metamorphosis of the red corpuscles, by its great affinity for oxygen and by its own proper metamorphosis, and not to form a product further serving for nourishment to the plasma.† There still remain the envelopes and nuclei of the red corpuscles. The envelopes are still too little known; but the nuclei have, in fact, a very close chemical resemblance to fibrin, so that their conversion into it (perhaps whilst oxygen is taken in and carbon given out) may be easily conceived.

§ 38. "The nuclei," Simon still continuing, "of the red corpuscles in the early stage of development are large and distinct, but as development advances they become smaller, and at last disappear—wholly, according to C. H. Schultz and Henle, in those corpuscles which are in their last stage. It is thus seen that the metamorphosis of the nuclei is by no means sudden, but goes on *pari passu* with the development of the red corpuscles. Burdach, R. Wagner, and Valentin are of opinion, that the red corpuscles, as long as they are circulating in the living body, have no nuclei, and that nuclei form only at the moment when the red corpuscles are removed

* Dr. S.'s Physiologische und pathologische Anthropochemie, is dated Berlin, April, 1842; but he mentions in the preface that he had enunciated the view in 1840, at the meeting of naturalists at Erlangen.

† The hematin he supposes is ultimately metamorphosed into hemaphæin or brown colouring matter of the plasma. This brown colouring matter of the plasma is continually being removed along with the urinary secretion, it being in fact the colouring matter of the urine also.

from the circulation. R. Wagner found that the mere contact with the atmospherical air determined the formation of the nuclei. According to this, the nuclei resemble chemically the fibrin of the plasma still more; and if it be unequivocally the case that in a red corpuscle in which the nucleus has, in consequence of advanced development, already become dissolved, its reappearance is observed when the red corpuscle has been withdrawn from the body, then, in my opinion, the conversion of the nucleus into fibrin would be sufficiently proved; as we know that no other constituent of the blood possesses the so characteristic property of being held in solution in living blood, and of being deposited as an insoluble mass by dead blood."

§ 39. To this opinion of Simon, that fibrin is derived from nuclei of the red corpuscles, an objection at once presents itself in the circumstance that there are *no nuclei* in the red corpuscles of man and the mammifera. But, waiving this objection, and admitting, with Simon, that nuclei do exist in the red corpuscles in their early stage of development, but that they soon disappear, being resolved into fluid fibrin; and that this fibrin is retained within the corpuscles and set free to be mingled with the plasma only when the red corpuscles themselves become dissolved. Admitting all this, it is quite clear that the red corpuscles of the blood of man and the mammifera, in the state in which they are usually found, should consist of fluid fibrin, in addition to their globulin, hematin, and envelopes.

§ 40. In support of the likelihood of this, Simon, as above seen, adduces the allegation which has been made by Burdach, Wagner, and Valentin, that the red corpuscles of the frog's blood, for example, present, while circulating within the body, no nuclei, and that the appearance of nuclei in the red corpuscles out of the body is owing to the fibrin contained in their interior coagulating.

§ 41. Supposing this allegation well founded as regards frogs' blood, there should, on the same principle, be found a nucleus in the red corpuscles of the blood of man and the mammifera after being drawn from the body, if there be fluid fibrin in their interior while circulating within the body; but as no nucleus presents itself, the conclusion is obvious, that there is no fluid fibrin in the interior of the red corpuscles while circulating within the body.

§ 42. But the allegation, that there is no nucleus in the red corpuscles of frogs' blood while circulating within the body, is not well founded. A nucleus does exist, although not distinctly seen, for the reason already given in the former Report. (§ 5.)

§ 43. The only fact tending to show that fibrin already exists in the red corpuscles, is one stated by Dr. Maitland, (an Experimental Essay on the Physiology of the Blood; London, 1838;) viz. that more fibrin is obtained by washing the coagulum of blood than by whipping. Maitland, believing that a nucleus exists and that it consists of fibrin, inferred from this observation, that the excess of fibrin was owing to the nuclei of the red corpuscles being retained within the coagulum. But, as already seen, there can be here, as regards human blood, no question of a nucleus. The additional quantity of fibrin must have been derived from fluid fibrin contained within the red corpuscles. But it may be doubted whether the excess in the product obtained by washing the coagulum over that obtained by whipping, were really fibrin. Did it not consist of the red corpuscles deprived, by washing, of their colouring matter—therefore of globulin and envelopes—retained in the meshes of the coagulated fibrin of the plasma?

§ 44. All that has now been argued appears to lead to the conclusion, that, in the red corpuscles of human blood, there is no fibrin, or at least no fibrin separate and distinct from the globulin, as long as the red corpuscles exist as red corpuscles. If fibrin have its source in the red corpuscles, therefore, that source must be the globulin, hematin, or envelopes. The hematin and envelopes, however, may be dismissed from consideration, for the reasons adduced by Simon.

§ 45. In regard to the globulin, Simon adduces two different kinds of arguments against the likelihood of its being the source of the fibrin in hyperinosis of the blood. His first argument is, that the quantity of fibrin produced is too disproportionate to that of the red corpuscles which have disappeared. His second argument is, that there is a more likely way in which the globulin is disposed of, viz. that it is the immediate source of urea, uric acid, and bilin.

§ 46. It would be out of place here to enter upon any consideration of the second argument: it is enough to remark, that it does not appear to carry much force with it.

§ 47. As to the first argument, it is to be replied, that, though the quantity of fibrin is, in the normal state of the blood, small, and the quantity of red corpuscles very large, it is only what might be expected, seeing that the red corpuscles may be viewed as a store whence fibrin is constantly being drawn, in a certain quantity, but which, on an emergency, might yield more than that quantity. There is no reason to suppose that, under ordinary circumstances, more red corpuscles disappear than may be actually accounted for by the quantity of fibrin which is produced. As fibrin is constantly being abstracted for the purposes of nutrition and secretion, the quantity produced must not be estimated by the quantity found at any one time in the blood. In inflammation, however, it is different, and the difficulty raised by Simon more striking; for here there is a sudden disappearance of a large quantity of red corpuscles, and a substitution of but comparatively a small quantity of fibrin, without there being any reason to suppose that an increased expenditure of fibrin has taken place in nutrition, secretion, &c. but rather on the contrary.

§ 48. If, however, the quantity of red corpuscles stagnant in the inflamed part, and the quantity of fibrin which may have been withdrawn from the blood by exudation be taken into consideration, the difficulty is lessened, if it does not vanish, especially when it is remembered that, in the production of the fibrin, a larger quantity of corpuscles must be expended than there is of fibrin produced, as it is the globulin only of the corpuscles which is supposed to be converted into fibrin, not the envelopes —in regard to the chemical nature of which, as already said, little is known. (§ 37.)

§ 49. Simon finds no chemical difficulty in supposing that a portion of globulin may be converted into albumen. There appears to be as little chemical difficulty in the supposition that globulin is converted into fibrin, seeing that all are equally protein compounds. But, as to the conversion of globulin into albumen, it is to be remarked, that it would be a retrograde instead of a progressive metamorphosis.

§ 50. It has been above mentioned (§ 25,) that, according to Mulder, it is not true fibrin but a *binoxide* and a *tritoxide* of protein, together comprehended under the name of *oxyprotein*, of which the buffy coat and of course the spontaneously coagulable matter of the plasma of the blood in hyperinosis are composed.

§ 51. Oxyprotein, Mulder affirms, is not derived from the albumen of the blood, but results from the oxydation of the fibrin by absorption of oxygen in respiration.

§ 52. Mulder here says nothing of the red corpuscles, nor of the source of the increase in the quantity of fibrin, which, by oxydation, should yield the excess of oxyprotein which exists in the blood in inflammation, over the quantity of fibrin and oxyprotein which exist in the blood in the natural state. But it is easy to supply this omission, on the principle contended for in this Report, in regard to the production of fibrin or oxyprotein, by supposing either that the red corpuscles are first resolved into fibrin, and that this fibrin, by absorbing oxygen, is, in conformity with Mulder's views, converted into oxyprotein; or that the red corpuscles absorbing oxygen, their globulin is at once resolved into oxyprotein.

§ 53. The hypothesis, that the fibrin of the blood is the result of the secreting action of the red corpuscles, was objected to by Dr. Carpenter in his Report on the Origin and Functions of Cells, above referred to. His principal argument against it is founded on the very facts which were adduced and relied on by the author of this Report as being most strongly in its favour—facts which, in the preceding pages, have been shown to have been previously made use of by the late Dr. Simon *on the same principle*, in support of nearly the same view.

§ 54. The facts referred to are: the diminution in the quantity of red corpuscles which is found to accompany hyperinosis of the blood, and the opposite inverse relation in the quantity of red corpuscles and fibrin in hypinosis of the blood.

§ 55. In employing the fact of the inverse relation in quantity between the red corpuscles and the fibrin in hyperinosis and hypinosis of the blood as an argument against the view, that the fibrin of the blood is the result of the secreting action of the red corpuscles, Dr. Carpenter entirely loses sight of the principle of the doctrine of secretion which he gives an account of a few pages further on in the same Report.

§ 56. But, besides the fact of the inverse relation in quantity between the red corpuscles and the fibrin, Dr. Carpenter adduces "another important fact having the same bearing," viz. the observation made by Remak, that "when an animal is bled largely and repeatedly, the quantity of red corpuscles in its blood is greatly diminished, whilst the proportion of the colourless corpuscles is increased." And this observation of Remak he couples with the circumstance, that it has been ascertained by Andral's investigations that the quantity of fibrin is but little affected by bleeding. The inference which Dr. Carpenter draws from all this is, that the white, rather than the red corpuscles are the agents of the elaboration of fibrin.

§ 57. It is quite true, not only that, when the same individual has been subjected to several bloodlettings, the red corpuscles become greatly diminished in number after each successive one, but also that the fibrin is increased in quantity. This is shown, in a very striking manner, by the following table from Andral and Gavarret:

| *Fibrin.* | *R. Corps.* | *Fibrin.* | *R. Corps.* | *Fibrin.* | *R. Corps.* | *Fibrin.* | *R. Corps.* |
|---|---|---|---|---|---|---|---|
| 6·3 . | 130 | 6·1 . | 123 | 4·0 . | 111 | 5·6 . | 133 |
| 7·7 . | 106 | 7·2 . | 120 | 5·5 . | 107 | 5·5 . | 131 |
| 8·2 . | 112 | 7·8 . | 112 | 6·5 . | 101 | 9·1 . | 128 |
| 9·3 . | 103 | 10·2 . | 101 | 9·0 . | 83 | 9·4 . | 102* |

§ 58. This diminution in the quantity of red corpuscles and increase in the quantity of fibrin after bloodletting is, however, still quite in accordance with the view, that the fibrin results from the solution of the red corpuscles. And though the proportion of colourless corpuscles was found by Remak† increased, his cases afford no ground for Dr. Carpenter's inference, that the white rather than the red corpuscles are the agents of the elaboration of fibrin, seeing that in these very cases of Remak the quantity of fibrin was diminished—a circumstance which Dr. Carpenter does not mention. Remak's cases, in fact, which Dr. Carpenter couples with Andral's cases of moderate bleeding, belong to the category of the cases mentioned by Andral, in which, when bleeding has been carried beyond certain limits, both the red corpuscles and the fibrin are found diminished—a state of the blood to which Simon gives the generic name of *spanæmia*, (*αἷμα* and *σπανος*, or *σπάνιος*, i. e. poverty of the blood.)

§ 59. As, in employing the fact of the inverse relation in quantity between the red corpuscles and the fibrin in hyperinosis and hypinosis of the blood, as an argument against the view, that the fibrin of the blood is the result of the secreting action of the red corpuscles, Dr. Carpenter entirely loses sight of the principle of the doctrine of secretion, on which the view is founded, so, when he appeals to an alleged increase of colourless corpuscles occurring along with an increase in the quantity of fibrin in the blood, as an argument in favour of the view he proposes, he equally loses sight of the principle of the doctrine of secretion.

§ 60. But suppose for a moment that his line of argument were just: let the facts which he alleges in support of it be examined. In the first place, it is obvious that, according to Dr. Carpenter's principle, there ought to be much fibrin in all cases in which it is alleged there exists, either normally or abnormally, a large quantity of colourless corpuscles. Do Dr. Carpenter's *facts* show this?

§ 61. Dr. Carpenter appeals first to the observation of Remak, that the colourless corpuscles were increased in number in the horse, after being largely and repeatedly bled. But it has been above seen that, in referring to this observation, Dr. Carpenter overlooks the circumstance, that Remak found the proportion of fibrin diminished; whilst, in referring to Andral to show that the quantity of fibrin is increased after bloodletting, he also overlooks the circumstance, that Andral says the same as Remak, that, after copious bleeding, the fibrin is diminished in quantity as well as the red corpuscles.

* Beyond certain limits, however, it was found by Andral that both red corpuscles and fibrin diminish.

† Medizinische Zeitung d. Ver. f. Heilk. in Preussen, 1841. N. vi, 7 Juli, p. 127.

§ 62. Dr. Carpenter next adduces the blood of the invertebrata as a fluid in which the elaboration of albumen into fibrin must be taking place, for the nutrition of the tissues, as in the higher animals; although there appears to be a total absence of any red corpuscles resembling those of the blood of the vertebrata: the globules contained in the fluid bearing, as suggested by Wagner, a much stronger resemblance to the corpuscles of the chyle.

In regard to this, it is to be remarked that, considering how very imperfect the knowledge of the blood of the invertebrata is as yet, any analogical argument drawn from it in the present case is of too loose a nature to merit the slightest attention.

§ 63. Dr. Carpenter further says, on the authority of Mr. Gulliver, that in very young embryos of vertebrata the white globules are nearly as numerous as the red particles; but omits to show the coexistence of fibrin in a correspondingly large quantity.

§ 64. Dr. Carpenter continues, "We should expect to find in the blood of the fœtus, up to the time of birth, a large proportion of white corpuscles; and this circumstance has been noticed by Dr. Barry, (and communicated to me by him,) with regard to the blood of the umbilical vessels of the placenta, which he had the opportunity of examining very shortly after its expulsion."

§ 65. Here again Dr. Carpenter makes no attempt to show the coexistence of a corresponding quantity of fibrin. But what are the facts of the case? Denis, in his chemical examinations, found the blood of the umbilical artery richer in red blood-corpuscles than the blood of the mother. Thus, in the blood drawn from a vein of the mother, there were of blood-corpuscles 139·9 per 1000; whilst, in the blood from the umbilical artery, there were of blood-corpuscles 222·0 per 1000! The quantity of blood-corpuscles in a dog three months old, was 97·0 per 1000; whilst, in a puppy one day old, it was 165·0 per 1000. As to the microscopical appearances presented by the blood of the umbilical vessels, in a case in which these vessels were carefully secured for the purpose of examining the blood, the author of this Report found the red corpuscles in great abundance—the colourless corpuscles in no preponderance.

§ 66. As to the quantity of fibrin in the blood of the fœtus and young animal, it is, according to Denis, small, in comparison with that of the red corpuscles; whilst in the mother it was as 2·4 to 139·9, it was in the umbilical artery as 2·2 to 222·0. Again, whilst in the dog three months old it was as 2·4 to 97·0, it was in the puppy one day old as 2·0 to 165·0!

§ 67. Dr. Carpenter continuing, says, "the presence of colourless corpuscles, sometimes to a large amount, in the blood of reptiles has long been known." True, but what is the amount of fibrin in the blood of reptiles?

§ 68. The existence of fibrin in chyle, though in small quantity, and when coagulated, much less consistent and firm than in blood, might be adduced in favour of the view, that the colourless corpuscles elaborate fibrin, seeing that there exist in the chyle colourless corpuscles and no red ones.

§ 69. In answer to this, it is to be remarked, in the first place, that the chyle, when it presents any marked quantity of fibrin, has been already mixed with the contents of lymphatics, and these are known to contain fibrin. In the second place, any fibrin which the chyle may present while within the lacteals in the early part of their course, may be supposed to be derived from the blood circulating in such large quantity in the villi during absorption. The fibrin presented by the chyle in the lacteals after they have passed through the glands, besides being derived from admixture of lymph from anastomosing lymphatics, the author of this Report agrees with Simon in supposing it to be derived from the liquor sanguinis of the blood circulating in the vessels of the glands. That the fibrin of the chyle, says Simon, (p. 245,) is formed from the constituents of the food, is not very probable.

§ 70. In the second place, according to Dr. Carpenter's principle, there should be a large quantity of colourless corpuscles where there is much fibrin. But as he has failed to show an increase of fibrin in the cases which he adduced, more or less correctly, as examples of the blood abounding in colourless corpuscles, so from what was above said (§§ 26-31), it will have been seen that here also he fails in proving an increase of colourless corpuscles along with an increase of fibrin.

§ 71. Lastly, Dr. Carpenter refers to the circumstance stated by Wagner, that the number of colourless corpuscles was found to be greater in the blood of well-fed frogs just caught in the summer season, than in those that had been long kept without food, and in those examined during winter.

§ 72. This, however, is nothing to the purpose. In the first case, the blood being in its healthy state, has a due proportion of fibrin, red corpuscles, and colourless corpuscles. The colourless corpuscles in proportion to the quantity of food taken, and the activity in the development of red corpuscles; the latter in proportion to the quantity of fibrin. In the second case, it is to be remarked, that the frogs were in a state spanæmia, or poverty of blood : the red corpuscles were few in number, in consequence of their gradual expenditure in the formation of fibrin, on the one hand, and their non-reproduction, on the other. The colourless corpuscles were few in number; those transformed into red corpuscles not having been duly replaced, in consequence of the want of food. The fibrin was in small quantity, having been expended in nutrition, and not duly replaced, in consequence of the now diminished quantity of red corpuscles. The non-reproduction of these again being owing to the fewness of the colourless corpuscles.*

§ 73. Dr. Carpenter's facts have thus been found not to warrant the inferences he would draw from them, even admitting the justness of his line of argument. But this line of argument has itself been shown to be contrary to the principle of the doctrine of secretion, which even Dr. Carpenter himself advocates.

§ 74. But how stands the relation between the colourless corpuscles and the fibrin of the blood ?

In the first place, as to chemical nature : Nothing is yet exactly known of the chemical nature of the colourless corpuscles of the blood.

In the second place, as to quantity : In healthy blood the quantity of colourless corpuscles may be stated to be, on an average, half a part in the 1000 parts of blood,† whilst that of fibrin is, as has been seen, according to Lecanu's average, three parts in the 1000. In inflammation at the time of hyperinosis of the blood, it remains to be shown that there is any increase in the quantity of colourless corpuscles; but suppose there is, how much higher a proportion does it bear to the increased quantity of fibrin than the half to three of the healthy state ?

§ 75. In fact, it may be said that the colourless corpuscles and fibrin, instead of being in a direct are in an inverse proportion to each other. This inverse relation, however, it is to be remembered, is not of the same sliding-scale kind as that between the red corpuscles and the fibrin, and therefore not to be viewed as an argument for the production of fibrin from the colourless corpuscles on the principle of the doctrine of secretion above recognized as the correct one.

§ 76. Thus, if the red corpuscles be found small in number when the fibrin is in large quantity, this is owing to the former having been reduced below their usual quantity, and the latter, *pari passu*, increased above its usual quantity. Whereas, if the colourless corpuscles be found few in number in proportion to the quantity of fibrin, this is no more than what already exists, even before any change in the quantity of fibrin has occurred.

§ 77. When, on the other hand, the colourless corpuscles are found in great number at a time when the fibrin is in small quantity, it will be found that the red corpuscles are at the same time in small quantity—that the blood is in the state of spanæmia; and that the relation which the large quantity of colourless corpuscles has, is not like that which the red corpuscles have with the small quantity of fibrin in hypinosis; but the relation is with the diminution in the quantity of red corpuscles themselves.

* Total privation of food, in the case of frogs, it may be remarked, is no more than defective supply of food in the mammifera. In these latter, the result of total privation may be compared to an acute disease. In dogs and horses kept for some days without food, the blood presents a great increase of fibrin and a corresponding diminution of red corpuscles. This Andral and Gavarret explain by the inflammation of the stomach which is found to have occurred.

† From an average of ten observations and enumerations made by Mr. T. H. Huxley, a distinguished pupil of the class of Anatomy and Physiology at the Charing-Cross hospital.

§ 78. The increase in the number of colourless corpuscles appears to be to yield a supply of red ones, in order to make good the loss of those which have disappeared. In fact, according to Remak's observations on the blood of the horse, as also Gulliver's, the red corpuscles are reproduced by being formed within the colourless ones, and become free by their solution. However this may be, the observation of Remak regarding the increase of the colourless corpuscles after repeated bleeding, which Dr. Carpenter quotes so partially, as above seen, to favour the view that fibrin is formed from the colourless corpuscles, Remak adduces to show that the red corpuscles are reproduced from the colourless ones.

§ 79. Lastly, when there exists along with an increase in the quantity of fibrin and a decrease in the quantity of red corpuscles, an increase in the quantity of colourless corpuscles, it will be found that the increase in the quantity of colourless corpuscles has taken place subsequently to the time when hyperinosis occurred; when the increase in the quantity of fibrin having been at the expense of the red corpuscles, the increase in quantity of the colourless is for the reproduction of the red corpuscles.

§ 80. Thus, then, if the red are reproduced from the colourless corpuscles, it is quite correct, in one sense, to say that the fibrin is derived from the colourless corpuscles. The rapidity with which red corpuscles disappear at the time fibrin is increased in quantity, or, as the author of this Report would say, are resolved into fibrin, and the comparative slowness with which they are reproduced from the colourless corpuscles will account for the relation between the fibrin and the colourless corpuscles not being of the same sliding-scale kind as that between the fibrin and red corpuscles.

§ 81. *Intimate nature of the process leading to hyperinosis of the blood in inflammation.* Considering it as having been by the preceding arguments, rendered very probable that the red corpuscles of the blood are the source of fibrin in the natural state, and of oxyprotein in hyperinosis of the blood, and that, of the constituents of the red corpuscles, it is the *globulin* which is converted into the fibrin and oxyprotein, an attempt may now be made to trace the intimate nature of the process leading to hyperinosis of the blood in inflammation.

§ 82. According to Mulder, the protein oxides always occur in the blood, being the matters employed in nutrition and secretion; but in inflammation they exist in a much greater quantity than in the healthy state. They are formed by oxidation of fibrin in the lungs; hence he says that in inflammation, in which the quantity is so much increased, there goes on a more active process of oxidation, this being the process leading to hyperinosis of the blood.

§ 83. That the process leading to hyperinosis of the blood is one of increased oxidation, is chemically true; but if what has been above said (§ 52) be well founded, there is along with the chemical process an increased activity of organic development. This is the view taken by Simon and the author of this Report.

§ 84. The author of this Report, in his Observations on the Blood, in this Review, for October, 1842, whilst he remarked, in opposition to Liebig's view of the red corpuscles being carriers of oxygen, that there is no reason to suppose that the liquor sanguinis less readily absorbs and is less a carrier of oxygen than the red corpuscles—a view which is the same as that advocated by Mulder, from his experiments, viz. that the fibrin oxydized in the lungs is the principal if not the only carrier of oxygen of the air—also remarked, that the absorption of oxygen by the red corpuscles might be looked upon as accessory to some peculiar function performed by them, rather than as being solely for the purpose of distributing the oxygen to the different parts of the system. That function the author of this Report considers to be the formation of fibrin, as above seen.

§ 85. Under the influence of the oxygen taken in during respiration, the red corpuscles run through their stages of organic development, and at last become dissolved. In this process, as in other cases of cell development, chemical changes occur—the globulin is converted into fibrin, the hematin into hemaphæin.

§ 86. In inflammation there is reason to believe that this process of organic development of the red corpuscles, in consequence of increased absorption of oxygen, takes place with increased activity. The red corpuscles, as already said, become dissolved in increased quantity, the globulin being converted into fibrin to be afterwards oxidated, or perhaps at once converted into oxyprotein.

The expenditure of oxyprotein in nutrition or in exudation at the inflamed part does not keep pace with its production by solution of the red corpuscles—hence its increased quantity; and the reproduction of the red corpuscles does not keep pace with their solution—hence their diminished quantity.

§ 87. *What are the circumstances in inflammation which determine the changes in the blood just described?* That the production of hyperinosis stands in some direct connexion with the local inflammatory process, and is not the consequence of supervening febrile excitement, may be inferred from the fact ascertained by Andral and Gavarret, that in the pyrexiæ there is hypinosis instead of hyperinosis, but should a local inflammation supervene in the course of a fever, that hyperinosis takes place.

§ 88. According to this, instead of inflammatory fever being a cause of the change in the blood, the change in the blood might perhaps be viewed as the cause of inflammatory fever.* To this it has been objected, that there are cases—cynanche tonsillaris, for example — in which fever distinctly precedes any local symptoms; but is not this premonitory fever, altogether different from that which supervenes after the local inflammation has developed itself?

§ 89. Supppose that the process of hyperinosis goes on, in part at least, in the vessels of the inflamed part, it might be said that the ordinary nutritive process being suspended, the oxygen of the stagnant blood, instead of acting on the tissues, acts on the red corpuscles themselves, and determines their conversion into oxyprotein. This is received into the circulating mass—perhaps in part exuded—and the place of the red corpuscles which were resolved into it is supplied by others stagnating; the cause of the inflammation continuing in operation.

§ 90. On this hypothesis the increased heat of the inflamed part seems to be readily explicable, especially when it is considered that in the red corpuscles there is an increased and constant supply of fuel†

### TERMINATIONS OR EVENTS OF INFLAMMATION.

Inflammation terminates either in mortification or in the healing process.

### I. NATURE OF MORTIFICATION.

§ 91. Mortification is the death of the part affected, together with the blood stagnant in it, and the matter which may have been exuded.

§ 92. The proximate cause or the condition on which mortification immediately depends, is complete stoppage of the circulation; either from all the vessels of the part being the seat of the inflammatory stagnation, or sometimes together with pressure exerted by the exuded matter, the operation of the pressure being favoured by the mechanical conformation of the part.

§ 93. The dead part being the same as a foreign body, the organization tends to throw it off. This separation is effected, as Dr. Billing remarks, by softening of the dead part from decomposition. It not being the object of this Report to enter further into the subject of mortification, the reader's attention is now requested to the *healing process.*

### II. NATURE OF THE HEALING PROCESS.

§ 94. In the course of the healing process, epiphenomena may occur, which, on account of their prominence and practical importance, have been spoken of as separate and distinct terminations or events; but the inflammation may not terminate on their supervention, and it is obvious that in the abstract they are merely species of the healing process and this though their tendency may sometimes be to interrupt rather than promote the cure.

§ 95. With this explanation, *resolution, adhesion, suppuration,* and *granulation,*

* Watson's Lectures on the Practice of Physic, vol. i, p. 171.

† Compare further on this point Simon's Anthropochemie, p. 182; and Williams's Principles of Medicine, § 437 et seq.

are to be looked upon as different species of the healing process, in which inflammation may terminate. Only it is to be remarked in regard to resolution, that it being the immediate and direct process by which the healing of inflammation takes place, it is also truly a *termination* of inflammation.

§ 96. Inflammation itself, it is known, may, by virtue of the species of the healing process in which it tends to terminate, act as a means of cure of some other disease. Again, one species of the healing process may counteract the wrong direction which another species is taking. And lastly, the termination in mortification itself, may come in as a curative process.

§ 97. As stagnation and exudation are the essential parts of inflammation, so the essential objects of the inquiry into the healing process of inflammation are how the circulation is reestablished in the part, and what becomes of the exuded matter.

### III. MICROSCOPICALLY-VISIBLE PHENOMENA ATTENDING THE REESTABLISHMENT OF THE CIRCULATION.*

§ 98. If the stagnation has not already existed long, and if the apparent fusion of the red corpuscles is not to any great degree, reestablishment of the circulation is observed to take place in the following manner. Oscillation of the agglomerations of corpuscles within the vessels occurs, such as was observed at the commencement of the stagnation. The red corpuscles resume their individual distinctness, and are disposed readily to become detached from each other, so that at every stroke of the heart, some are forced into neighbouring anastomosing vessels in which the circulation is going on, and are carried along in the stream. Sometimes the whole of the corpuscles within a vessel, are seen to be thus at once set afloat.

§ 99. If the stagnation has already existed for some time, and if the apparent fusion of the red corpuscles is in a great degree, reestablishment of the circulation does not so readily ensue. In this case the red corpuscles are not observed to become again distinct individually, but the agglomerations of them having become loose in the vessels, and being at every stroke of the heart made to protrude a little into the neighbouring vessels into which they open, and in which the circulation is going on, pieces of them are detached in the form of irregular flocculi which are carried along in the stream of the circulating blood, where the red corpuscles appear to be at last separated from each other, and to become individually distinct, being at the same time restored to their natural collapsed state, by the action of the plasma.†

§ 100. Though the circulation has thus become reestablished, the vessels may be observed still to have an unusual quantity of colourless corpuscles accumulated on the inner surface of their walls, so that in comparison with their natural caliber, but a very small stream of blood is allowed to thread its way along.‡

§ 101. Besides colourless corpuscles, some red ones also continue here and there adhering to the walls of the vessels, but still pale and distended. At length, however, they may be seen to become detached, and to be carried along in the stream.

§ 102. The absorption of exuded matter, and the disappearance of the red tinge of the adjacent parenchyma which had resulted from the transudation of hematin eventually ensue.

§ 103. Lastly, it is some time before the dilated vessels fully recover their usual caliber.

### IV. EXPLANATION OF THE MICROSCOPICALLY-VISIBLE PHENOMENA ATTENDING REESTABLISHMENT OF THE CIRCULATION.

§ 104. In the first place it is to be observed that though the vessels may be found still dilated, this is not to be assumed as an indication that nervous influence has not

* See Hastings, *ut supra*, and especially Emmert, *ut supra*.

† Sometimes as reestablishment of the circulation occurs at one place, a new stagnation makes its appearance at another.

‡ This circumstance of the reestablishment of the circulation in vessels in which a large accumulation of colourless corpuscles still exists would alone, in the absence of other reasons, be sufficient to show the unfounded nature of Dr. Williams's opinion, referred to in last Report, that accumulation of colourless corpuscles originally determines the stagnation of the red ones.

been again restored to them. The overstretched fibres are unable all at once to contract as usual; and as regards the capillaries, they have not recovered their elasticity weakened by over distension.

§ 105. The reestablishment of the circulation may be said in a general way to depend on the cessation of the disposition which the red corpuscles have to remain aggregated, and to adhere to the walls of the vessels, in consequence of which the red corpuscles now yield to the *vis a tergo*.

§ 106. As to the cause of this cessation of the disposition of the red corpuscles to remain aggregated, and to adhere to the walls of the vessels: If the opinion as to the immediate cause of the stagnation of the blood enunciated in the former Report, §§ 75, 76, be well founded, it may be naturally inferred that the return of nervous influence to the walls of the vessels is the cause sought for. When, however, the changes in the stagnant blood are taken into consideration, (§§ 1, 2, 4, 6, 9,) it must be admitted that besides restoration of nervous influence, some other cause is in operation.

§ 107. As the apparent fusion of the red corpuscles which supervenes more or less quickly on stagnation, even when the mass of blood is healthy, is like that which is presented by the red corpuscles of buffy blood, and appears to be owing to the action of plasma inspissated as regards its protein constituents, but deprived of its salts by exudation of serum, (§§ 4, 6, 9,) so the additional cause sought for, may perhaps be found by comparing with the behaviour of the red corpuscles in the process of reestablishment of the circulation that presented by the red corpuscles of buffy blood out of the body, after the coagulable plasma has been by their close aggregation pressed out from among them, viz., that the red corpuscles have then lost much of their disposition to remain aggregated, as is shown by the little cohesion of the under part of the clot. This, without going minutely into the matter, may be said to be owing partly to the abstraction of the coagulable plasma from among the red corpuscles, and partly to the direct action on them of the serum, which is by and by separated. The addition of a saline solution at the same time that it causes collapse of the distended corpuscles, disposing them to separate.

§ 108. To apply the inference which may be drawn from the comparison now made in explanation of the process of reestablishment of the circulation in inflammation. In consequence of the abstraction of coagulable plasma from among the red corpuscles stagnant within the vessels by exudation, and the reabsorption of the serum into the obstructed vessels, (§§ 7, 8,) the red corpuscles are disposed more readily to separate from each other, and from the walls of the vessels, and thus to yield to the *vis a tergo*.

§ 109. Hence, as the explanation offered by Henle of the immediate cause of stagnation was rejected as such, (see former Report,) but adopted (in the present Report) as applicable to the production of the apparent fusion of the red corpuscles, (§ 9,) so the explanation which Henle offers of the apparently fused state of the red corpuscles, viz. reabsorption of serum into the obstructed vessels, (§ 7,) whilst it is rejected as such, (§ 8,) is here admitted to play a part at least in the process of reestablishment of the circulation.

§ 110. The explanation which Henle himself offers of the process of the reestablishment of the circulation is this: "Whilst the tendency of the vessels to contract returns, the plasma on the one hand must acquire the normal state, or at least one the opposite of the inflammatory, so that the red corpuscles readily separate from each other again; the impulse of the arteries on the other hand becomes stronger, on account of the contraction." (Bericht, &c., p. 162.)

§ 111. According to Emmert, "by the exudation of a part of the stagnant and changed blood, (which exudation is known to have occurred by the red colour of the adjacent parenchyma,) the mass of the contents of the vessels becomes less, and the cohesion of it appears therefore to become weaker, for close to where this mass meets by anastomoses with the circulating blood, it is seen to be readily detached and carried away."

§ 112. The probability that some of the stagnant red corpuscles may dissolve, has been above admitted (§§ 12, 89); but unless the conditions above considered (§§ 105-7)

were in operation, this solution could not contribute to the reestablishment of the circulation. The cause of the inflammation continuing, the vessels would be refilled by stagnating blood as fast as they were emptied. This, however, would explain the long continued exudation which sometimes goes on at one place.

§ 113. The fate of the exuded matter* constitutes, as above mentioned, one of the essential objects of the inquiry into the nature of the healing process of inflammation, but before proceeding with this inquiry, it is necessary to consider more particularly than has yet been done, the exuded matter itself.

### V. EXUDED MATTER.

§ 114. Exudation takes place either into the interstices of the parenchyma, or on surfaces natural or raw. In the case of a natural surface, its epidermis, or its epithelium if it be resisting, is raised up into a blister by the exuded matter.

§ 115. As already said, the exuded matter from being at first serous, comes at last to contain a greater or less quantity of fibrin or fibriniform matter, (oxyprotein,) and in this state is a clear viscid fluid, usually called lymph.†

§ 116. As this fluid is the same as the plasma of the blood, it of course has the same properties, physical and chemical. It may remain fluid or coagulate, and having coagulated, the serum may or may not be separated. In the latter case, the exuded matter is gelatiniform, in the former the coagulum is consistent or diffluent, according to the proportion of fibrin or oxyprotein contained in the exuded matter. The serum which is separated, may either be soon removed by absorption or collect.

§ 117. Examined microscopically, the recently-exuded matter appears quite amorphous, without any trace of organization, except that when coagulated, it may be more or less indistinctly fibrous, and covered with oil-globules, appearances which, however, have nothing in common with the organization which afterwards ensues.

§ 118. The corpuscles which the fibrinous matter very soon after exudation is found to contain, have been alleged to be the colourless corpuscles of the blood, which have escaped from the vessels ; but this is not the case. As already said, none of the corpuscles of the blood pass out along with the exuded fluid as long as the vessels are entire.

§ 119. The corpuscles in exuded matter are new formations developed after exudation, developed in it in fact, as in a blastema.‡

### VI. ABSORPTION OF EXUDED MATTER.

§ 120. If much matter has not been exuded, absorption of it takes place, as the circulation in the obstructed vessels becomes reestablished, and this the more readily if the exuded matter is still fluid.

### VII. DEVELOPMENT OF ORGANIC ELEMENTS IN THE EXUDED MATTER.

§ 121. When absorption does not take place, the exuded matter becomes organ-

* See, on this subject, Vogel's article 'Entzündung,' in Wagner's Handwörterbuch der Physiologie, p. 341 et seq. ; and Henle's 'Bericht.'

† The exudation of lymph from a wounded surface was found by Dr. John Thomson, to take place in four hours after the wound ; by Sir A. Cooper, in six hours in dogs, and twelve or fourteen in man.

‡ The colourless corpuscles observed still accumulated on the inner surface of the walls of the vessels in which the circulation has become reestablished, (§ 100,) appear to be the same as those referred to by Mr. Travers in his recent work on Inflammation, under the name of lymph-globules or particles. The organic elements formed in the exuded matter as in a blastema above spoken of, (§ 119,) appear to be what Mr. T. describes as the separated lymph-particles, and which he thinks are the material of new structures. They are, he says, separated subsequently to the effusion of liquor sanguinis. The deposit effused with the liquor sanguinis which is on the contrary an amorphous exudation, and serves merely for the intermedium of organization in a close apposition of surfaces (being separated from the liquor sanguinis with which it is effused,) would not serve as a base for the new solid ; it is soon absorbed, being only a temporary band or adhesive layer in harmony with the parts, though serving the important purpose of consolidation by anastomosis of the contiguous vessels of opposite sides, or union by the first intention.

ized; in it, as in a blastema, organic elements are formed. The process by which this takes place is altogether the same in principle as that by which the normal development is originally effected. At the same time that the exuded matter becomes changed morphologically, it is changed chemically; thus, for example, it may now yield gelatin (pyin), no trace of which, Mulder affirms in opposition to Bouchardat's statements, is found in the buffy coat.

§ 122. There are three principal ways in which the exuded matter has been found to be developed and disposed of.

1. After having attained a certain development, the organic elements are, if formed in the interstices of a parenchyma, dissolved and removed by absorption, or if formed on a surface, thrown off.

2. The organic elements undergo further development into tissues, homologous or heterologous.

3. The organic elements formed in the exuded matter are nucleated cells, pus-corpuscles, which undergo no further development, but are discharged along with the fluid in which they are suspended, (liquor puris,) in the form of pus from the body, in greater or less quantity.

§ 123. *First form of organization of exuded matter.* The elements in respect of form, which are in this case developed in the exuded matter are the "filled cells" of Henle,—the "granule-cells" of Vogel,—a certain stage in the development of which is represented by the "compound inflammation globules" of Gluge.

§ 124. According to Vogel, the granule-cell is nucleated. It is at first from 1-3375th inches to 1-1125th inches, and gradually grows until it reaches the size of 1-900, 1-675th inches. The contents of the cell are at first in small quantity. Afterwards the cell contains a large quantity of small dark granules, like elementary or pigment granules, so that from being at first transparent and colourless, the cell afterwards becomes quite opaque, from the colour of its contents, assumes even a brown or black tint, and looks like an aggregation of granules, the nucleus, and frequently also the cell-wall being quite imperceptible. The addition of water or acetic acid, however, by distending the cell wall, brings it into view.

§ 125. Acetic acid at last dissolves the cell-wall, but leaves the nucleus and granules. Ammonia or potass dissolves both cell-wall and nucleus, but not the granules. These, however, are in general soluble in ether, whence it would appear that they are composed of fat.

§ 126. When by the action of acetic acid, the wall of the cell is dissolved, and the granules scattered, the nucleus comes into view. It is distinguished by its size, granular appearance, and generally by the existence in it of a nucleolus.

§ 127. According to Vogel, the granule-cells are capable of no further development, but after reaching their full size, and becoming quite filled with granules, they begin to undergo a retroceding metamorphosis. The nucleus and cell-wall disappear, the granules remaining, and for a time still held together by a connecting substance; in this stage constituting the compound inflammation globules. Ultimately they become completely separated from each other.

§ 128. After this breaking up of the granule-cells, the whole exuded matter originally present is converted into a semifluid pappy mass, consisting of the separated granules suspended in the serum of the originally exuded plasma, in which state it is readily absorbed, the serum first, and lastly, the granules after being dissolved.

§ 129. Henle gives a different view of the succession in the stages of development, and retroceding metamorphosis of the organic elements under consideration.

The organic element in the state of the so-called "compound inflammation globule," which is an agglomeration of minute semitransparent granules held together by an amorphous gelatiniform mass, but without any membranous envelope, he supposes to be the first stage, and to result from elementary granules aggregating together. In the second stage the agglomeration of granules becomes surrounded by a membrane; whence a granule-cell results. The granules then dissolve gradually from within outwards, leaving only a few to represent the nucleus.

§ 130. Granule-cells may be formed along with pus-corpuscles in the matter exuded, on an inflamed surface, e.g. a mucous membrane in catarrh, and may be thrown off entire. Bühlmann* correctly considers the compound inflammation globule as identical with the *corpuscula granulosa* found in the sputa in chronic catarrh, and which is composed of an aggregation of corpuscles which he calls *albuminous corpuscles.* What Bühlmann describes as *melanotic cells* appear to be the same in the *stage of granule-cells,* the granules being dark. Many of such cells are found in the sputa, without the granules being dark.

§ 131. Within these cells as they occurred in the sputa of a catarrh when going off, and which were of various sizes, some as large as 1-300th of an inch, the author of this Report observed smaller nucleated cells covered over and imbedded in the granules. These smaller cells varied in number from one or two to a great number, according to the size of the parent cell. This observation, of the correctness of which he repeatedly satisfied himself, recalled to his mind the development of cells from the embryo cell of the ovum, with which is connected the division and subdivision of the yelk. It is however to be remarked that in the latter case, the embryo cells metamorphose the yelk, in order to the development of the embryo, whereas the formation of cells within the granule-cells leads to nothing.

§ 132. *Second form of organization of exuded matter.* This consists in the conversion of the exuded matter into tissues. The process by which this is effected is in general altogether the same in principle as that by which the tissues are originally developed. It would be beside the purpose of this Report, to enter into any inquiry as to what tissues are capable of being regenerated from matter exuded in inflammation, still more would it be beside the purpose of this Report, to enter into any inquiry regarding the different modes in which different tissues are regenerated.

§ 133. Regeneration of epithelium or epidermis, of cellular tissue, and of capillary vessels, is all that it is necessary to direct attention to here.

§ 134. *Epithelium or epidermis.* The development of new epithelium or epidermis, in other words the skinning over of a sore on a mucous membrane or on the skin, is too well known to require more than mention here.

§ 135. *Cellular tissue.* When the cell-theory was first promulgated, it was supposed that both in the original genesis, and in the regeneration of cellular tissue from exuded matter, perfect nucleated cells required to be first formed, and that these cells by farther development, gave rise to fibres ; Henle has, however, shown in his 'General Anatomy,' p. 198, that in the original genesis of the cellular tissue, perfect nucleated cells are not required, and he now expresses it as his opinion, that although nucleated cells round or elongated into flat fibres, are always met with where new cellular tissue is being formed, the principal mass of the fibres is developed in the following manner.

§ 136. There are formed numerous round nuclei, (exudation corpuscles,) in the cytoblastema, which elongate, becoming slender, and arrange themselves one after another in rows. At the same time the cytoblastema is resolved into flat fibres from 1-5600th to 1-3700th of an inch broad. On the surface of these flat fibres lie the nuclei which become partly absorbed, partly coalesce, to form nucleus and elastic fibres. The broad fibres either remain at this stage of development, in this state resembling the fibres of the middle coat of the arteries, or they split into finer fibrils, begin to curl and become true cellular tissue.

§ 137. Mr. Gulliver, appealing to the fibrous structure which fibrin presents immediately on coagulation, has been led to express doubts as to the universality of the application of Schwann's doctrine. Mr. Gulliver is quite correct when he says that he could never see any satisfactory evidence that the fibrils of fibrin are changed cells. Direct observation clearly shows that the fibrin is at once formed into fibres in the act of coagulating. But Mr. Gulliver is not correct in adducing the fibrous structure of fibrin as an argument against the development of fibres from cells.

§ 138. It has been above shown with Henle, that Schwann's theory required limitation, but the following remarks are given by Henle to serve as an answer to Mr.

* Beiträge zur Kenntniss der kranken Schleimhaut der Respirationsorgane und ihrer Producte durch das Microscop, p. 52, Bern, 1843.

Gulliver's question, "What is the proof that these fibrils, (of coagulated fibrin,) may not be the primordial fibres of animal textures ?"

"In most cases the softening and solution of coagula, by means of which they are rendered fit for absorption, or changed into pus, depends on a process of organic metamorphosis, which at its commencement is not to be distinguished from reorganization. The first step of the process, or at least a change accompanying it, is the disappearance of the retiform fibrillation, if such was present. Without the coagulum losing its consistence, and often at a time when the new microscopical globular elements are first deposited in separate groups, those fibres become finer, paler, and at last invisible. They have thus no direct share in the formation of new tissues. To this I might admit one exception. The fibres of the striped coat of the arteries, and of the heart which lies immediately underneath the epithelium, or when this is wanting, which forms the free inner surface of the above-mentioned organs, resemble so closely in form and arrangement the fibres of fibrin, that we cannot refrain from supposing they both have a similar origin. In the fully-formed state the fibres of the striped coat are broader and darker, and do not change in acetic acid ; they are, however, met with also narrow, pale, and often scarcely to be distinguished. Admitting that the fibres of a fibrinous lamella have in consequence of some chemical change, lost their solubility in acetic acid, (as is the case with epithelium-cells,) we have a lamella of the striped vascular coat. It is especially by the production of numerous layers of this membrane, that the morbid thickening of the arterial walls is produced which precedes the so-called atheromatous degeneration. Whether the fibre which yields the material for this purpose is derived directly from the blood circulating in the vessels themselves, or exuded from the *vasa vasorum* is as regards this question, indifferent." (Bericht, p. 177.)

§ 139. *New vessels.* It is here proper to remark, in the first place, that it is a not uncommon though a very erroneous notion, that many of the vessels which for the first time become visible in an inflamed part—the conjunctiva for instance—at the commencement of inflammation are new productions. Not only is this not the case, but even in an advanced stage of an inflammation, when exudation has taken place, it may be that no new vessels are formed, although the exuded matter undergoes development into other structures.

§ 140. On this point Dr. Hastings, more than twenty years ago, recorded the following results of his observations (ut supra, pp. 93-4): "In the foregoing experiments, if inflammation began and terminated without any lesion of the part affected, new vessels were never formed. After the most attentive scrutiny, the author is induced to believe, that the apparent formation of new vessels is always caused by numerous capillaries becoming dilated, and carrying an arterial red blood. In all cases, however, in which inflammation arises from wounds, a whitish matter seems to be deposited by the divided vessels, when they are much dilated and full of an arterial red blood. After this matter has been deposited some time, small vessels can be seen in it, which communicate with the inflamed capillaries of the incised part. Thus, through the medium of these new vessels, a slow circulation is at first effected ; but by degrees the recently formed substance becomes more vascular, and then a free communication ensues between the capillaries of the opposite edges of the wound ; and the newly deposited matter at length assumes an appearance nearly similar to the rest of the web."

§ 141. Before considering this question further it is also proper to remark, that what is often meant by "organization" is the development of new vessels ; but as has been already said, organization of exuded matter may take place quite independently of the development of blood-vessels. The development of new vessels, in fact, is in itself a process of organization which presupposes the development or organization of other tissues. New vessels are not formed for the purpose of "vitalizing" "effusions of the organizable materials of the blood," for such effusions are already vitalized. It is from such effusions that the new blood-vessels themselves are developed, and that along with the development or organization of other tissues, such as the cellular. The blood-vessels are formed in order to fetch and carry away the materials concerned in the nutrition and further development of these tissues.

§ 142. All the best observations on the development of new vessels hitherto tend to the establishment of the proposition that it takes place in this way. At the same time that the new cellular or other tissue is being developed cells are formed, which coalesce with and open into each other, and form networks of capillary vessels, at first quite separate from and independent of the old vessels of the part with which they only afterwards enter into communication, as Mr. Hunter supposed.

§ 143. The opinion, so generally entertained, that the new vessels are merely prolongations of the old, appears to have arisen from the circumstance that, in the ordinary modes of examination, the new vessels have not been recognized except in advanced stages of development, when they have already formed a communication with the old; for, until this communication be established, the new vessels cannot be injected, either naturally or artificially. This also explains the apparent suddenness with which new vessels are formed.

§ 144. But it is to be remarked, that what has been sometimes taken for new vessels may be referred, as Henle points out, to a deceptive appearance, viz., to blood effused into the interstices of the network of fibrinous fibres, the red corpuscles of which blood have agglomerated into rolls, and these again into branched and retiform figures. Henle thinks that the appearances described by Andral, Hasse, Skoda, and Kolletscha, as rapid vascularization and formation of blood in pleuriditic exudations, may have been of this nature.

§ 145. The newly developed tissues having served a purpose which was merely temporary, may undergo a retroceding metamorphosis in order to be absorbed, as in the preceding case. As examples of this, may be mentioned provisional callus uniting fractured bones, the new vessels formed in the cornea, &c.

§ 146. *Third form of organization of exuded matter.—Suppuration.* The elements, in respect of form, which are in this case developed in the exuded matter, are *pus-corpuscles.* Pus corpuscles are globular cells measuring, on an average, 1-2250th of an inch in diameter, finely granulated on the surface, and sometimes presenting an indistinct appearance of a nucleus. By the action of water or dilute acetic acid, the cell-wall becomes distended and transparent, so that the diameter of the corpuscle is increased, and the nucleus comes distinctly into view. And it is now seen as if composed of two or three, more rarely four granules, usually separate and distinct from though closely applied to each other. Sometimes, however, the nucleus granules are seen to be continuous and homogeneous throughout, forming one multilobed whole.

§ 147. Pus-corpuscles are suspended in a considerable quantity of the serum of the originally exuded plasma (*liquor puris*)—the whole forming a sort of emulsion-like fluid, well known under the name of *pus*.

§ 148. Pus is developed from matter exuded either in the interior of organs, in the cellular tissue, &c., or on the surface of organs or open wounds. In the first case the pus collecting forms an abscess, and in the second case there is a suppurating surface.

§ 149. Pus-corpuscles are not capable of any further development, but they, and the fluid in which they are suspended, are expelled from the body. The pus of an abscess in general tends to the surface, for the purpose of being evacuated. The matter which forms on a surface, either on the exterior of the body or the wall of a cavity communicating with the exterior, may be considered as already evacuated.

§ 150. In some cases, pus not being expelled from the body the liquor puris is absorbed, and the corpuscles becoming broken up are also eventually removed by absorption. The process, as described by Vogel, is this: When the pus is not artificially evacuated, and does not make a way for its own escape, it may undergo still further changes. The pus-corpuscles gradually collapse, the pus is converted (especially when the serum is absorbed) into a thick, grumous mass, or into a thin fluid mixed with greasy flocculi, and the mass, as well as the flocculi, appear under the microscope as a matter quite undefined, consisting of small granulary molecules, generally under 1-11250th of an inch in size, which are not to be distinguished from other dissolved and decomposed organic matters, such as, e. g., encephaloid or tuberculous mass, from organic detritus in general. In this state, but only in this, the pus is capable of being wholly absorbed.

§ 151. It is thus seen that whilst the granule-cells developed in matter exuded into the substance of organs are as a regular result broken up, dissolved, and absorbed, the pus-corpuscles developed in a similar situation tend to the surface, in order to be evacuated directly from the body, absorption occurring merely as a rare exception to this rule.

§ 152. But it is to be remarked that granule-cells are also sometimes found in pus, or, as above shown, in puriform sputa, and are in this case directly expelled from the body.

§ 153. *Genesis of pus-corpuscles.* According to Henle and Vogel, pus-corpuscles are formed in the exuded matter thus: In the exuded matter small bodies, sometimes single, generally joined together in twos and threes, first make their appearance. These are the nuclei. Around them the cell-wall is gradually formed. This cell-wall is at first delicate and quite transparent, but afterwards becomes finely granular, by which its transparency is impaired and its nucleus either rendered indistinct, or altogether concealed.

§ 154. There are differences of opinion as to whether the division of the nucleus above mentioned as being generally shown by the action of acetic acid be an early and progressing stage, or a late and retroceding stage. According to Henle and Vogel, it is an early and progressing stage. The small bodies seen applied to each other in twos and threes are elementary granules, which gradually coalesce to form a simple nucleus, but the earlier the stage of development, they are the more readily disjoined by the action of water or acetic acid.

§ 155. According to Vogel the blastema in which pus-corpuscles are formed is either the *already coagulated* fibrin of the exuded plasma, or the plasma with its fibrin *as yet uncoagulated* The first is the case in all pus-formations in the interior of organs, in the cellular tissue, and, in short, in all cases in which the exuded matter has time to coagulate before the commencement of organization. The second is the case in regard to pus which appears on the surface of organs, or in cavities which open externally, on all mucous membranes, on the surface of the cutis, in open wounds, &c.

§ 156. According to Vogel, when the pus-corpuscles are formed in a blastema of already coagulated fibrin they are at first observed here and there, as if imbedded in the amorphous or indistinctly fibrous blastema. Afterwards the whole exuded matter is gradually converted into pus-corpuscles. These, as soon as they have reached their full development, separate from each other and become mingled with the serum which had separated from the fibrin at the time of its coagulation. The pus-serum is the original blood-serum poured out along with the blood plasma, perhaps somewhat modified. It is only when the pus-corpuscles become mingled with it that fluid pus presents itself.

§ 157. It is to be believed that at the commencement of a phlegmonous abscess, pus is produced in the manner just described, but when some has already collected it is probable that what is subsequently formed is developed on the inner surface of the walls of the abscess, in the same way as pus on a suppurating surface, the plasma, as fast as it is exuded, being formed into pus-corpuscles.

§ 158. Suppuration on a mucous surface, for example, may be said to be an excessive but imperfect development of epithelium cells, and which are thrown off with morbid rapidity. The suppuration on a wounded surface may, to a certain extent, be viewed in the same light, the anormal epithelium in the form of pus being intended to protect the surface.* So also the formation of pus on the walls of an abscess (pyogenic membrane). The pyogenic membrane is a new vascular structure, somewhat analogous to granulations, developed from the exuded matter. The production of anormal epithelium in the form of pus, originally determined by the irritation of a foreign body, or of a bit of dead cellular tissue, or dead coagulated fibrin, is kept up afterwards on the surface of the pyogenic membrane by the irritation of the matter itself.

§ 159. *Conditions determining whether one or other of the above-described modes of development of the exuded matter shall take place.* Whether one or other of the

* In order to protect the new tender granulations, they are covered with Pus. (Billing's Principles of Medicine.)

above-described modes of development of the exuded matter shall take place, appears to depend on the quantity and quality of the exuded matter, and the place where it is deposited. These, again, depend on the nature, degree, and seat of the inflammation. The quality of the exuded matter will also, of course, depend on the condition of the blood, and this again will depend partly on the inflammation itself, and partly on the state of the constitution of the individual in whom the inflammation exists.

§ 160. As regards particular states of the constitution—Mr. Dalrymple* has called attention to "the rapid organization of lymph in cachexia;" meaning, by "organization," the development of new vessels, or vascularization.

§ 161. It is quite true that lymph exuded within the eye, for example, in inflammations occurring in cachectic states of the constitution—in syphilitic iritis, in scrofulous iritis—more frequently become vascular than in cases of iritis occurring in otherwise healthy subjects. But is this not explicable by the circumstance that in such cases as the former the inflammatory action subsides less quickly and perfectly, and the exuded matter is accordingly less readily absorbed than in cases of healthy inflammation?

§ 162. The circumstance that the lymph which is exuded in drops on the iris in syphilitic iritis at first yellow, afterwards assume a red hue more or less marked, is supposed by Mr. Dalrymple to denote organization, i.e. vascularization of it, but might not the redness be owing either to dissolved colouring matter, or, as in the cases above referred to, (§ 144,) to extravasated red corpuscles?

§ 163. *Is blood in substance capable of reorganization?* Mr. John Hunter was of opinion that it is, Dr. John Thomson adduced very good reasons against it, more lately Mr Dalrymple has published a case which he thinks is an example of it. Dr. Pirogoff also has published affirmatory observations, and on the question Professor Henle † remarks: "It is frequently, but incorrectly affirmed, that plastic exudations only, but not blood in substance, are capable of reorganization. The development of thrombus, of the corpora lutea in the ovaries, &c., is a decided contradiction to this opinion."

§ 164. In considering this question, it is to be remembered that to answer it in the affirmative it would not be necessary to demonstrate the existence of new vessels, though if *new* vessels did exist they would be a proof not only of organization, but of a somewhat high degree of it.

§ 165. The proofs of the organization of blood in substance, which have been adduced, have been principally the existence of blood-vessels, as evidenced by the blood coagula admitting of being injected. But in such a high degree of development or organization, the mass of the coagulum should have been itself found already converted into tissue—cellular tissue for example. Has this been proved to be the case? And supposing it to have been found to be the case, who will say that it was an example of blood in substance organized, rather than of exuded lymph coloured with effused blood?

§ 166. In his case Mr. Dalrymple‡ does not speak of the mass of the blood-clot otherwise than if it were still in the form merely of coagulated blood, though "minutely organized with innumerable new and minute vessels," whose arrangement was so determinate and uniform as to leave no doubt of the entire dissimilarity of the organization of this tissue, from the periosteum on the one hand and the bone on the other; in short, that though the larger vessels might be old, the rest were all new.

§ 167. If the mass of the clot was still blood in substance, Mr. Dalrymple endeavours to prove too much from the existence of the vessels. If, on the other hand, the mass of the clot was composed of tissue interwoven with blood-vessels, then the case is no proof of the organization of blood in substance; for, to repeat, Who will say that no lymph was originally exuded, that it was blood alone which was originally effused?§

§ 168. Pirogoff's observations, quoted by Henle,|| in support of the opinion that blood in substance is capable of organization, are as follow: "The new substance which unites the cut ends after section of the tendo Achillis is produced, according to Pirogoff,¶ from effused blood. If the section is so made that little blood is effused

* Med Chir. Trans. for 1840. † Bericht. ‡ Loc. cit.

§ Dr. Macartney says that he has succeeded in forcing an injection into the coagula formed in the cavities of the heart after death, which injection presented the appearance of red elongated lines? (On Inflammation, p. 51.)

|| Bericht, p. 210. ¶ Ueber die Durchschneidung der Achellessehne, Dorp. 1840, p. 18.

(from the skin towards the bones), the sheath of the tendon collapses, its walls grow together, the cut ends of the tendon become thick and clubbed, and the function is not reestablished. In some cases injection of blood into the sheath of the tendon, when performed immediately after the operation, served as a substitute for the natural bleeding." There is no proof here that the effused blood forms the new substance.

§ 169. In regard to the organization of the corpora lutea, the following are the conclusions to which the observations hitherto made by the author of this Report seem to point.

In the first place the matter, whatever it is, out of which the yellow body is developed, is effused into the interstices of the stroma of the ovary immediately outside the walls of the Graafian follicle. The vessels and fibres found in the yellow body, are not of new formation, but the original ones which connect the walls of the Graafian follicles with the stroma of the ovary, and which are stretched and separated by the effusion into their interstices of the matter whatever it is, from which the proper substance of the corpus luteum is developed.

§ 170. The corpus luteum may therefore be excluded from the question of vascular organization.

§ 171. As to the proper substance of the corpus luteum.—The microscopical elements of this have been found somewhat different in different cases. It has in one case been found composed of masses of elementary granules, and imbedded in them pale delicate cells having somewhat of the appearance of red corpuscles of the blood bleached and collapsed by the action of reagents, except that they were larger and nucleated. In another case along with the minute elementary granules, a considerable number of larger bodies, apparently of the same substance, and which resembled that of the nuclei of pus-corpuscles, for example. Some of these bodies seemed to contain nucleoli, and in several instances there was the appearance of a very pale cell-membrane around them, of about double the diameter of the red corpuscles of the blood. In a third case, besides the elementary granules, there were merely shapeless particles like shreds of broken-up cells.

§ 172. From such observations, coupled with the well-known fact that the corpus luteum at length disappears, the author of this Report has been led to the opinion that the matter exuded into the stroma of the ovary around the Graafian follicles, to form a corpus luteum, attains merely a certain degree of development, and then undergoes a retroceding metamorphosis, in order to be rendered fit for eventual absorption, as in the process described at §§ 127-8. The fibres and vessels in the yellow body being the same fibres and vessels which connected the outside of the Graafian follicle with the stroma of the ovary.

§ 173. The masses of elementary granules may be compared to granule-cells in the stage of the so called compound inflammation globules. As to the nucleated cells, they might be viewed as formations developed within the masses of elementary granules, in a manner similar to what is above described in regard to the granule-cells contained in the sputa of a fading catarrh. (§ 131.)

§ 174. Seeing that the corpus luteum does thus present a certain degree of organization, the point to be determined in reference to the question under consideration is whether or not the matter from which the corpus luteum is developed be blood in substance.

§ 175. The matter is, according to all observers, at first *bloody looking;* but the author of this Report is not aware if any microscopical observations have been made, showing that it is pure blood, and not fibrinous exudation mixed with blood, or showing that if at first pure blood, this does not give place to fibrinous exudation before organization commences.

§ 176. The only observation at all bearing on the point which the author of this Report has himself made is the following: A very large and recent corpus luteum. The appearance which a section of it presents to the naked eye, somewhat like that of a clot of blood. Here and there only a trace of the characteristic yellow appearance of the fully-formed corpus luteum can be detected. Membraneous bands composed of the vessels and fibres between the burst Graafian follicle and the stroma of the ovary, seen extending through the clot-like mass.

§ 177. As to the microscopical characters of this mass: It consists of granulous corpuscles like compound inflammation globules, closely aggregated, and some red blood-corpuscles interspersed among them. The granulous corpuscles appear of a yellowish red tinge, as if they had imbibed colouring matter from the red corpuscles which are somewhat bleached and shrivelled.

§ 178. From this it is certain that there had been blood effused; but the presence of perfect red blood-corpuscles cannot be admitted as a proof that the granulous corpuscles were formed from them. Were the granulous corpuscles formed from the fibrin of the plasma of the effused blood, or were they developed from exuded lymph, the presence of the red blood-corpuscles being owing to extravasation of blood occurring in addition to the exudation?

In other cases it has been sufficiently proved that the so-called compound inflammation globules are not formed, as Gluge supposed, from red blood corpuscles.

§ 179. The reestablishment of the circulation and the fate of the exuded matter having been considered, a review may now be taken of them in their correlations as they are exhibited in the different species of the healing process.

### VIII. RESOLUTION OF INFLAMMATION.

§ 180. When resolution of inflammation of a vascular part, visible externally, takes place, the pain and heat cease, the redness disappears, the swelling subsides, and the part is at last restored to its natural state.

§ 181. When resolution of inflammation of the cornea above referred to takes place, the irritability of the eye ceases, and, on the one hand, the congestion of the neighbouring conjunctiva and sclerotica diminishes; then, on the other, the exudation in the substance of the cornea disappears.

§ 182. Resolution of inflammation consists in the reestablishment of the circulation, and absorption of the exuded matter. The exuded matter may have been still fluid, and may not have become in any way organized, but it is probable that in many, if not most cases apparently ending in resolution (such as it has just been defined), the exuded matter has first become organized into granule-cells, as above described. These are then either thrown off from the surface on which they were formed; or if formed in the interstices of a parenchyma they are, by the process of retroceding metamorphosis, again rendered fluid in order to be absorbed. This constitutes what Vogel calls resolution of exuded matter.

§ 183. "It is," says Vogel, (p. 345) "especially observed after inflammations of *internal organs*,—the brain, lungs, spleen, liver, &c. In most chronic inflammations of the cerebral substance and inflammatory softenings of this organ there is formation of granule-cells. In all inflammations of the lungs in which the patients died from other causes after the occurrence of resolution, as ascertained by the general phenomena as well as by auscultation, I found this process. On external parts, in the cellular tissue, in muscles, on membranous organs, the formation of granule-cells as an event of inflammation is more rarely observed, perhaps, for the reason that there is seldom opportunity to examine carefully external parts after inflammation which has not passed into suppuration, but become resolved. After internal inflammation of the eye, in consequence of needle operations, in which the patient died from other causes some time after the operation, I twice found formation of granule-cells in the exuded matter poured out from the inflamed parts into the interior of the eye."

The author of this Report, as above shown, considers the *corpus luteum* of the ovary as a physiological example of a transitory formation of a similar nature.

### IX. HEALING OF A WOUND.

§ 184. *In the healing of a wound* conversion of the exuded matter into tissues takes place under two different kinds of circumstances. And according as one or the other obtains, there is what surgeons call "healing by the first intention" or by "adhesion," or "healing by the second intention" or by "granulation and suppuration."

§ 185. The stagnation and exudation, and therefore the redness and swelling of the part, do not cease on the occurrence of either of these processes. They continue until the healing process is accomplished, the stagnation, or at least a slow circulation in the part, being the necessary condition of exudation; the exudation again that of the supply of material wherewith regeneration is effected.

§ 186. In the last Report the cornea was referred to as a part which, from its non-vascularity, forms a good subject for observing, in a manner uncomplicated by the presence of the vessels in which the blood is stagnant, the development and disposition of the exuded matter in the healing process. The matter exuded into the substance of the cornea from the vessels of the adjacent conjunctiva and sclerotica may (as far as examination with the naked eye, or the eye assisted merely by a magnifying glass goes,) be seen to undergo the different modes of development above described.

§ 187. It may be seen that pustules form and that, in the healing of incisions and ulcers of the cornea, adhesion and granulation, and, lastly, cicatrization, may take place without the development of new vessels—a convincing proof that neither suppuration nor "organization" necessarily implies the development of new vessels.

§ 188. But, as hinted in the former Report, new vessels may be formed in the matter exuded into the cornea, and this again affords a very interesting and readily observable example of the development of new vessels, uncomplicated by the previous existence of other vessels in the part.

§ 189. Here the mode of development of new vessels, as described above (§§ 142-3), is to be recalled to mind, in order to guard against the very obvious though erroneous inference, that the new vessels are produced merely by the shooting of the old vessels of the conjunctiva, for example, into the cornea; the new vessels not being visible until, in consequence of an inosculation with the old, they become injected with red blood.

§ 190. The new vessels, having served their purpose, shrink and disappear, and it is not until this that cicatrization is completed. (§ 145.)

§ 191. *Healing by the first intention or adhesion.* In this case the matter exuded on the cut surfaces becomes forthwith, and all of it, converted into tissues—cellular tissue and capillaries—by which the divided parts are reunited. An epithelium or epidermis is then formed on the surface in the ordinary way, and cicatrization is completed.

§ 192. *Healing by the second intention, or granulation.* This is a slower process than adhesion. The inflammatory congestion persisting matter continues to be exuded. One part of it is converted into cellular tissue and capillaries—granulations are composed of these new tissues in process of development; another part is converted into pus, which, as above said, serves as a sort of epithelium to the granulations.

§ 193. As the healing approaches completion, the quantity of exuded matter converted into pus becomes less and less in comparison with that expended in the formation of tissues. At last, no more pus being formed, the exuded matter is developed into epithelium or epidermis, and cicatrization is in this case also completed. As this takes place the granulations contract and become less vascular by the shrinking and disappearance of many of the vessels which existed in them, as is so distinctly observable in the case of the cornea, in regard to all the vessels.*

§ 194. That the tissue of cicatrice is not quite homologous with the old tissue is very evident in the case of the cornea by the resulting opacity.

§ 195. The newly-formed tissues—whether their formation be by the adhesive process or by granulation—besides effecting union of divided parts, or supplying the place of lost parts (regeneration), may be formed in excess. If the tissues thus developed in excess be homologous, *hypertrophy* is the result; if heterologous, *tumours* of different kinds, *induration*, &c.

* The process going on in an ulcerating sore is the opposite of that going on in a granulating sore. For whereas, in the latter, the exuded matter is developed partly into tissues, partly into pus; in the former the exuded matter is not only not organized into tissues, but those at the surface of the part lose their vitality and are thrown off, as Mr. J. W. Earle (on the Nature of Inflammation, in Medical Gazette, vol. xvi, p. 254, London, 1835,) and Dr. Billing remark, in minute portions with the discharge, which is either a mere sanies, or at the most an imperfectly developed pus.

This death of the ulcerating surface is not owing to any destructive action of the exuded matter which constitutes the discharge, but is owing to the same condition which determines the death of the exuded matter itself. From this it is seen that *ulceration* belongs to the head of *mortification*.

